# Electroporation as a vaccine delivery system and a natural adjuvant to intradermal administration of plasmid DNA in macaques

**DOI:** 10.1038/s41598-017-04547-2

**Published:** 2017-06-23

**Authors:** Biliana Todorova, Lucille Adam, Slobodan Culina, Raphaël Boisgard, Frédéric Martinon, Antonio Cosma, Mart Ustav, Thierry Kortulewski, Roger Le Grand, Catherine Chapon

**Affiliations:** 1 CEA – Université Paris Sud 11 – INSERM U1184, DRF/Jacob/Immunology of Viral infections and Autoimmune Diseases (IMVA), IDMIT infrastructure, 92265 Fontenay-aux-Roses, France; 2 Vaccine research institute (VRI), Créteil, France; 3 CEA, Institute of Biomedical Imaging (I2BM), DSV/SHFJ/INSERM U1023, CEA, Orsay, France; 4 Institute of Technology, University of Tartu, Tartu, Estonia; 5 CEA, Photonic Microscopy Platform, Institute of cellular and molecular radiation biology (IRCM), Fontenay-aux-Roses, France

## Abstract

*In vivo* electroporation (EP) is used to enhance the uptake of nucleic acids and its association with DNA vaccination greatly stimulates immune responses to vaccine antigens delivered through the skin. However, the effect of EP on cutaneous cell behavior, the dynamics of immune cell recruitment and local inflammatory factors, have not been fully described. Here, we show that intradermal DNA vaccination combined with EP extends antigen expression to the epidermis and the subcutaneous skin muscle in non-human primates. *In vivo* fibered confocal microscopy and dynamic *ex vivo* imaging revealed that EP promotes the mobility of Langerhans cells (LC) and their interactions with transfected cells prior to their migration from the epidermis. At the peak of vaccine expression, we detected antigen in damaged keratinocyte areas in the epidermis and we characterized recruited immune cells in the skin, the hypodermis and the subcutaneous muscle. EP alone was sufficient to induce the production of pro-inflammatory cytokines in the skin and significantly increased local concentrations of Transforming Growth Factor (TGF)-alpha and IL-12. Our results show the kinetics of inflammatory processes in response to EP of the skin, and reveal its potential as a vaccine adjuvant.

## Introduction

Among the various vaccination approaches against infectious diseases such as human immunodeficiency virus (HIV), deoxyribonucleic acid (DNA) vaccines have several advantages: they are easily produced, provide opportunities for molecular engineering, lack anti-vector immunity, and have the potential to promote both cellular and humoral immune responses^1^. However, despite their high immunogenicity in murine models, DNA vaccines have shown poor efficacy in large animal models and humans^2^. New strategies to improve DNA vaccines include the optimization of transcriptional control elements and codons^3–5^, the use of adjuvants, such as Toll-like receptor (TLR) ligands^6^, cytokine expressing plasmids^7–9^ or apoptosis-based adjuvants^10–12^ and the choice of an appropriate delivery system such as local electroporation (EP)^13–15^.

In particular, EP has been largely used to enhance plasmid DNA uptake and increase the number of antigen-producing cells^16, 17^. In addition, EP modifies blood vessel permeability and facilitates leukocyte extravasation in the exposed area^18^. However, the effects of EP on cutaneous antigen presenting cells (APCs) and on the dynamics of cell recruitment at the vaccine site have not been fully described.

In a previous study, we demonstrated that intradermal (id) administration of the auxo-GTU^®^-multiHIV plasmid (GTU for Gene Transport Unit) combined with noninvasive EP induces a strong and persistent polyfunctional T-cell response in macaques^19^. Here, we investigated the early events that occur in the skin and the subcutaneous tissue after id vaccine delivery, which may be associated with strong immunogenicity. In particular, we studied the effect of EP on antigen expression, dermal and epidermal APC behavior, immune cell infiltration, epidermal damage and local cytokine production in the skin of macaques.

## Results

### Electroporation stimulates local antigen expression, especially in the epidermis

**Figure 1:**
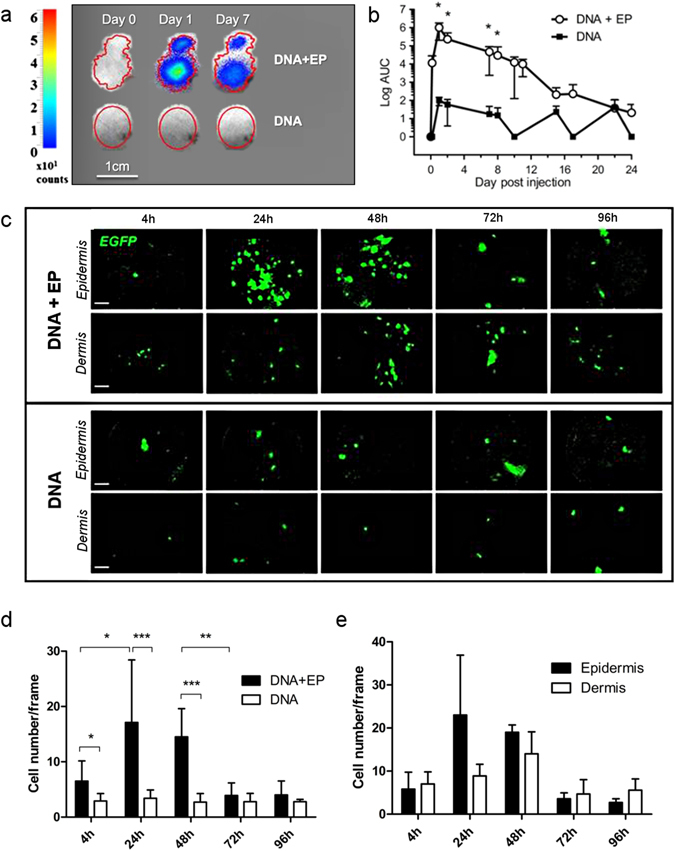
*In vivo* imaging of vaccine antigen expression at the site of injection. (**a**) Representative *in vivo* bioluminescent images of luciferase expression in macaque skin at day 0, 1 and 7 after intradermal injection of auxoGTU^®^-luc-EGFP ± EP. (**b**) Quantification of luciferase expression after vaccination with (n = 10) or without electroporation (n = 3). Mann-Whitney test. (**c**) *In vivo* fibered confocal microscopy showing EGFP expression by auxoGTU^®^ in the epidermis and the dermis after vaccination ± EP. Scale bar: 100 µm. (**d**) Quantification of EGFP^+^ cells from 4 to 96 h after vaccination, from 10 different frames. Paired and unpaired t-test. (**e**) Distribution of EGFP^+^ cells in the epidermis and the dermis after DNA vaccination with electroporation. Data are presented as mean ± SD; *p < 0.05; **p < 0.01; ***p < 0.001; EP, electroporation.

We used *in vivo* bioluminescence and fibered confocal fluorescence microscopy to monitor the expression of antigens in the skin after the id injection of DNA vaccine with or without EP. When EP was applied, luciferase was expressed for up to two weeks after the injection of auxo-GTU^®^-Luc-EGFP plasmid with a peak at 24 h, but was poorly expressed (*p* = 0.014) after immunization without EP (Fig. 1a,b). This observation was confirmed by the direct visualization of cells expressing EGFP: there were significantly more EGFP^+^ cells at 4 h (*p* = 0.0183), 24 h (*p* = 0.0002) and 48 h (*p* = 0.0002) after id vaccination with EP than after id vaccination with DNA alone (Fig. 1c,d). Furthermore, antigen expression at 24 h seemed to be mainly located in the epidermis (Fig. 1c,e).

### Langerhans cells are highly mobile, interact with transfected cells and leave the epidermis after DNA injection with EP

**Figure 2:**
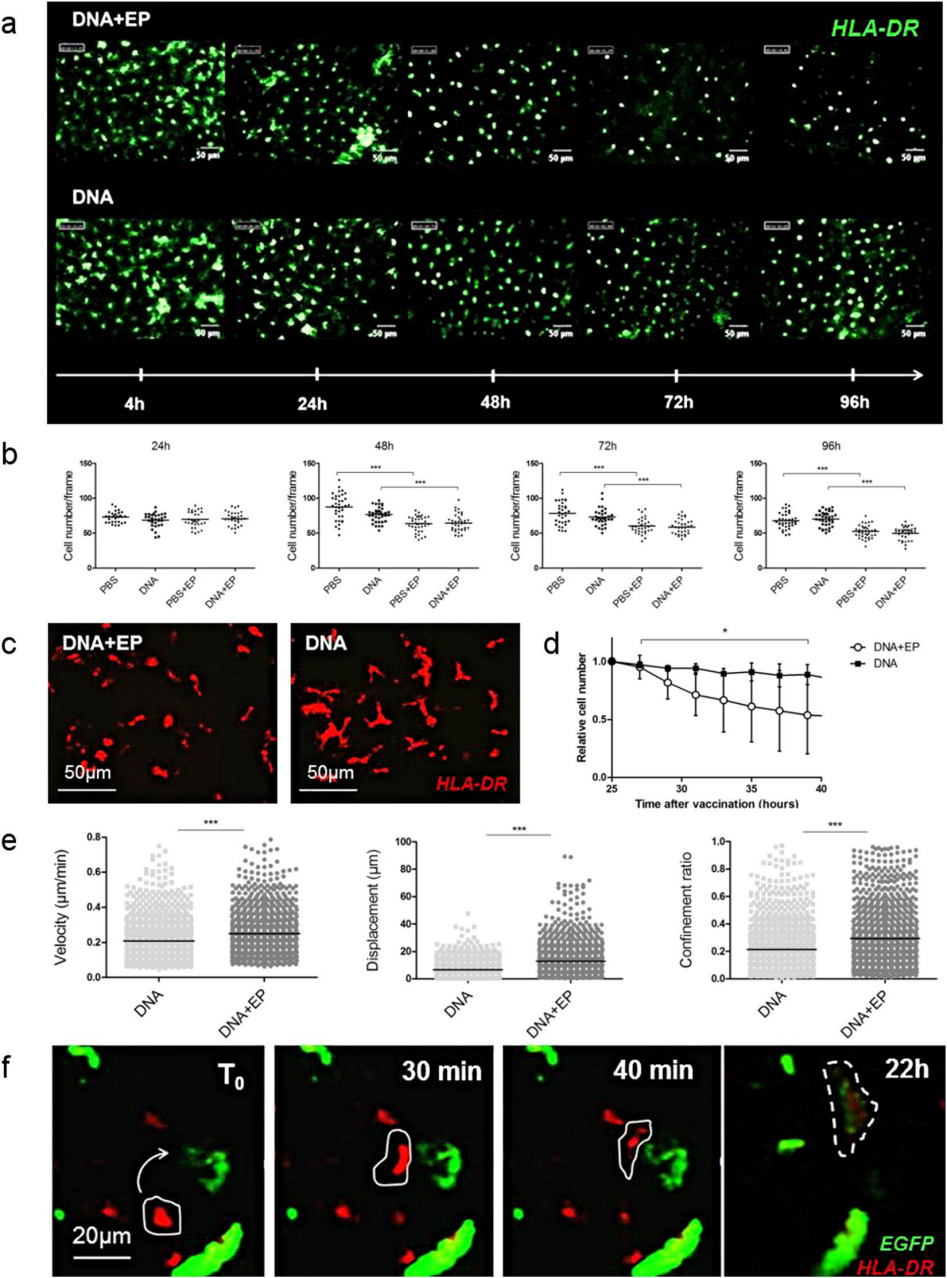
Behavior of LCs at the site of vaccination. (**a**) HLA-DR labeled LC network was visualized by *in vivo* fibered confocal microscopy and (**b**) quantified at the site of injection. Kruskal-Wallis test. *Ex vivo* confocal videomicroscopy of HLA-DR-labeled LC, showing (**c**) LC morphology and (**d**) the variation of the LC number normalized to the cell number at the first time point (n = 3). Friedman test. (**e**) LC motility parameters were measured from three independent experiments. Each point represents one cell. Mann–Whitney test. (**f**) *Ex vivo* confocal videomicroscopy of the epidermis 24 h after vaccination with the auxoGTU^®^-Luc-EGFP vector with EP. Arrow indicates APC displacement toward an antigen expressing cell. Dotted line shows a HLA-DR and EGFP co-stained LC. Data are presented as mean ± SD; *p < 0.05; ***p < 0.001; EP, electroporation.

Fluorescent stained epidermal cells, after id injection of HLA-DR antibody (Ab), were organized into a homogeneous network with dendritic morphology and were considered as Langerhans cells (LCs). These cells were imaged *in vivo* for 5 days after DNA ± EP in three independent experiments (Fig. 2a). Their number remained stable between 24 h and 96 h after the injection of plasmid DNA or PBS without EP (Fig. 2a,b). Interestingly, LC density was significantly decreased from 48 h after EP (*p* < 0.0001), suggesting their migration from the epidermis (Fig. 2b). LCs were imaged continuously for 18 h in skin explants taken 24 h after vaccination, to assess their behavior and motility. EP was associated with morphological changes of LCs, including dendritic retraction and rounding up (Fig. 2c), which corresponds to an activated LC profile^20^. EP also led to LC migration from the epidermis because their number decreased significantly (*p* = 0.017) over time (Fig. 2d). Furthermore, the velocity and displacement of LCs were significantly higher (*p* < 0.0001) after EP than in non-electroporated skin, and EP was associated with a high confinement ratio, illustrating the directional movement of LCs after vaccination with EP (Fig. 2e). In addition, some LCs interacted for several hours with antigen producing cells (EGFP^+^ cells), resulting in double stained cells, which suggests antigen capture by LCs (Fig. 2f).

### EP causes immune cell infiltration in the skin and promotes the mobility of recruited APCs in the dermis post-vaccination

**Figure 3:**
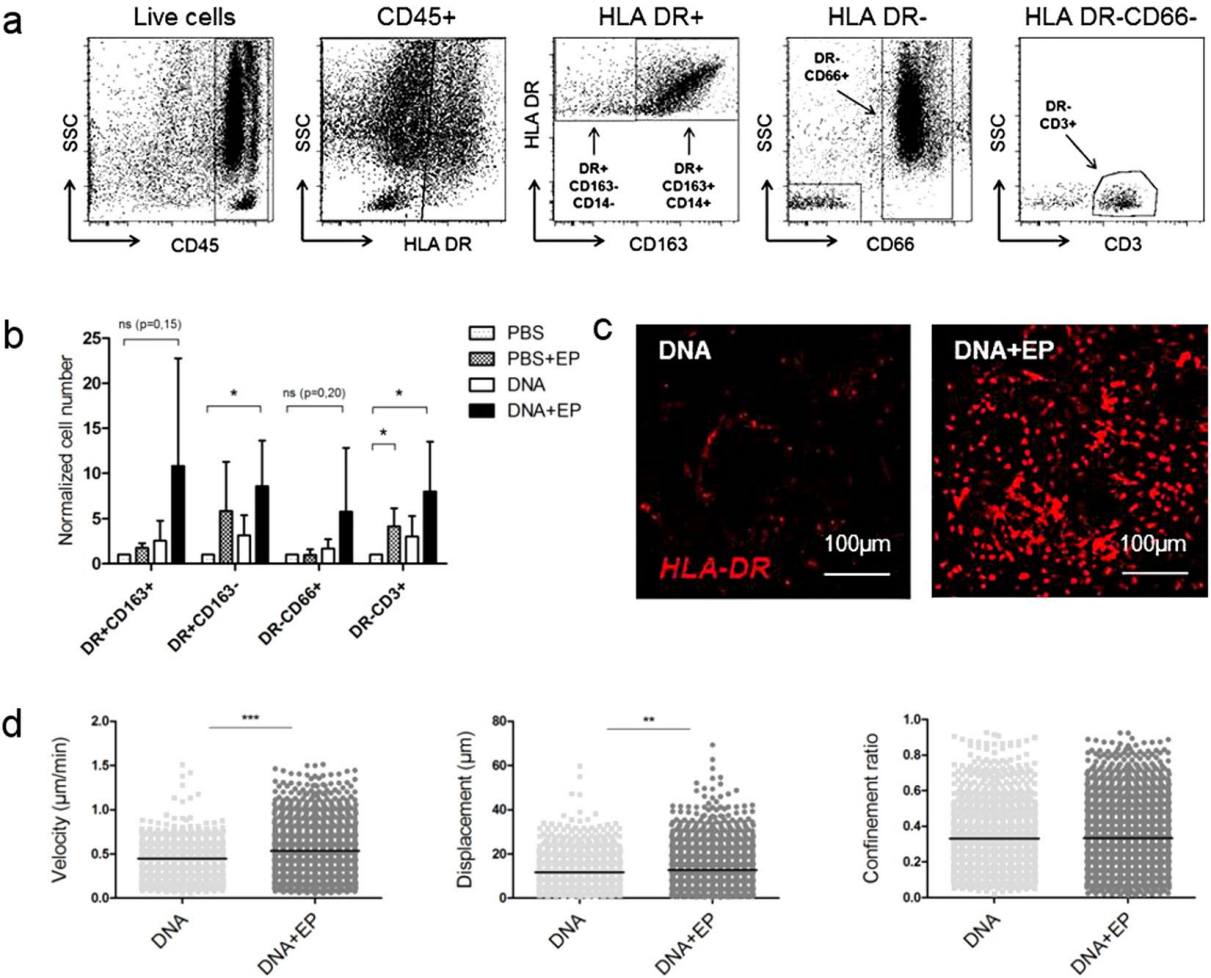
Characterization of immune infiltrates in the skin after EP or DNA + EP. (**a**) Skin cell suspensions were first gated for their morphology and viability. The gating strategy is shown on the skin of immunized macaque. Four cell populations of interest were analyzed: DR+CD163+CD14+ (monocytes/macrophages), DR+CD163−CD14− (dendritic cells), DR−CD3+ (T lymphocytes) and DR−CD66+ (neutrophils). (**b**) Flow cytometry analysis of skin cell recruitment 24 h after vaccination (n = 5); cell number was normalized to absolute cell number after PBS injection. Friedman test. (**c**) Representative *ex vivo* confocal videomicroscopy images of HLA-DR labeled dermal APCs 24 h post-injection of DNA ± EP. (**d**) Analysis of dermal APC motility 24 h post-vaccination. Each point represents one cell (n = 3). Mann–Whitney test. Data are presented as mean ± SD; *p < 0.05; **p < 0.01; ***p < 0.001; EP, electroporation.

Flow cytometry revealed the presence of four leukocyte populations (CD45^+^ cells) in the skin (both epidermis and dermis) (Fig. 3a). EP without DNA injection was able to induce lymphocytes T CD3+ infiltration in the skin 24 h after treatment (Fig. 3b). Furthermore, when EP was associated to the administration of DNA vaccine, the number of dendritic cells and T lymphocytes was significantly higher in electroporated skin 24 h after immunization than in skin injected with PBS alone (*p* = 0.0226 and *p* = 0.0055). There was a similar trend for monocytes/macrophages (*p* = 0.15) and DR^−^CD66^+^ neutrophils (*p* = 0.20) (Fig. 3b). We also observed this cell recruitment in the dermis by confocal microscopy, which revealed the presence of many HLA-DR^+^ cells 24 h after DNA injection with EP (Fig. 3c). In contrast to the epidermis, the dermal APC confinement ratio was not modified by EP whereas cell velocity (*p* < 0.0001) and displacement (*p* = 0.0022) were still significantly higher in electroporated skin than in non-electroporated skin (Fig. 3d).

### DNA injection combined with EP affects deep skin layers

**Figure 4:**
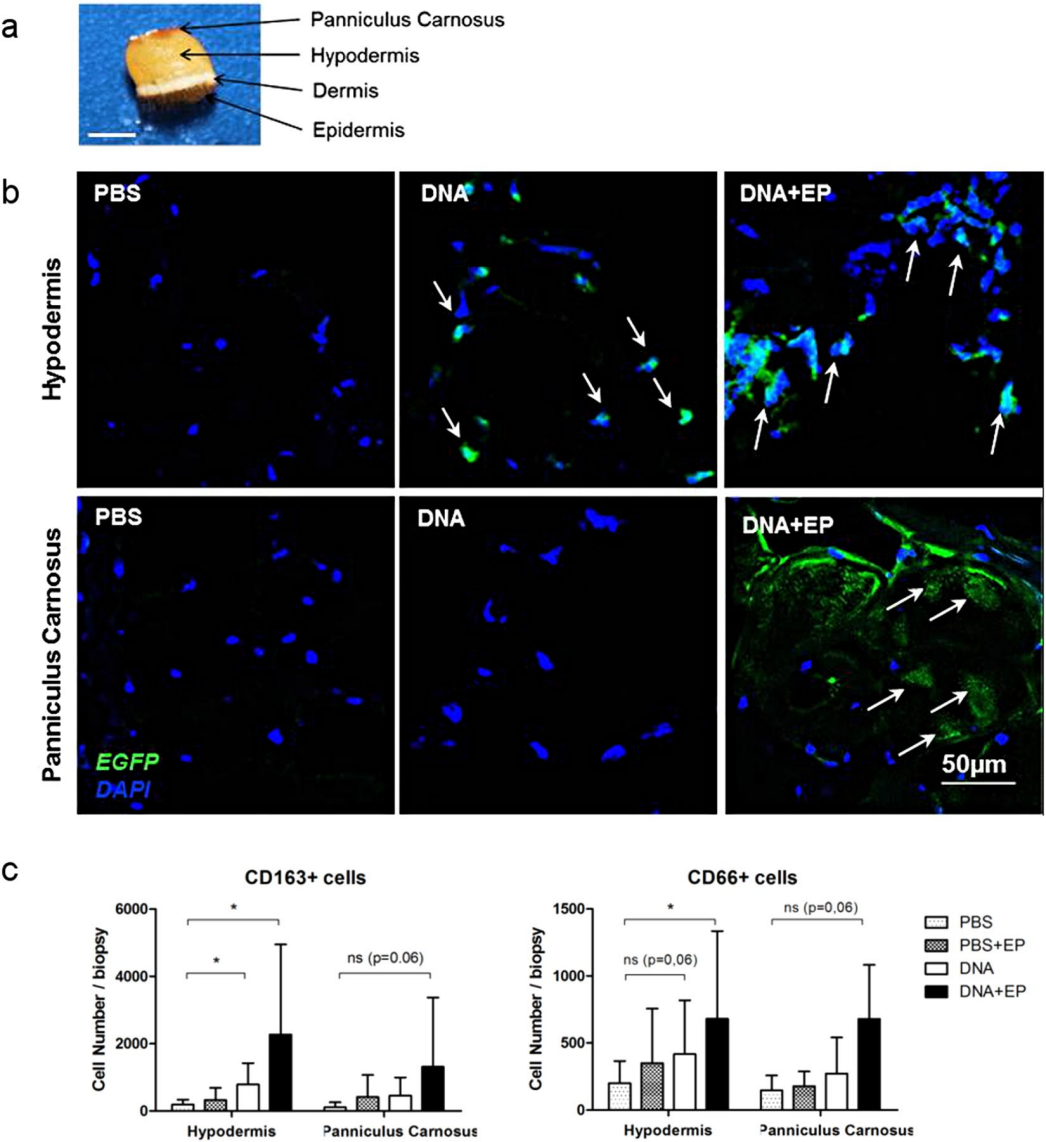
Antigen expression and immune cell characterization in the subcutaneous tissues. Six animals were injected with PBS or auxoGTU^®^-Luc-EGFP with or without EP and skin biopsies were analyzed 24 h post-vaccination. (**a**) Macaque skin biopsy showing the four different layers. Scale bar: 5mm. (**b**) Vaccine antigen was detected on frozen skin sections with a polyclonal anti-GFP-AF488 antibody. Arrows show EGFP expressing cells. (**c**) Flow cytometry analysis of DR+ CD163+ (macrophages) and DR−CD66+ (neutrophils) cells extracted from the hypodermis and the cutaneous muscle (panniculus carnosus). Data are presented as mean ± SD; *p < 0.05: Wilcoxon test. EP, electroporation.

Because the hypodermis and the subcutaneous muscle are interconnected and localized under the skin (Fig. 4a), we investigated the effect of EP on these skin layers. We detected antigen production in hypodermal cells after the id injection of plasmid DNA with or without EP (Fig. 4b, upper panel), and found that both DR^+^CD163^+^ and DR^−^CD66^+^ cells infiltrated this tissue (Fig. 4c). By contrast, we could detect antigen expression in subcutaneous muscle only after DNA administration with EP (Fig. 4b, lower panel). In this condition we observed a tendency of DR^+^CD163^+^ and DR^−^CD66^+^ cells infiltration at the site of immunization (*p* = 0.0625) (Fig. 4c).

### Vaccine antigen expression co-localizes with areas of damaged keratinocytes

**Figure 5:**
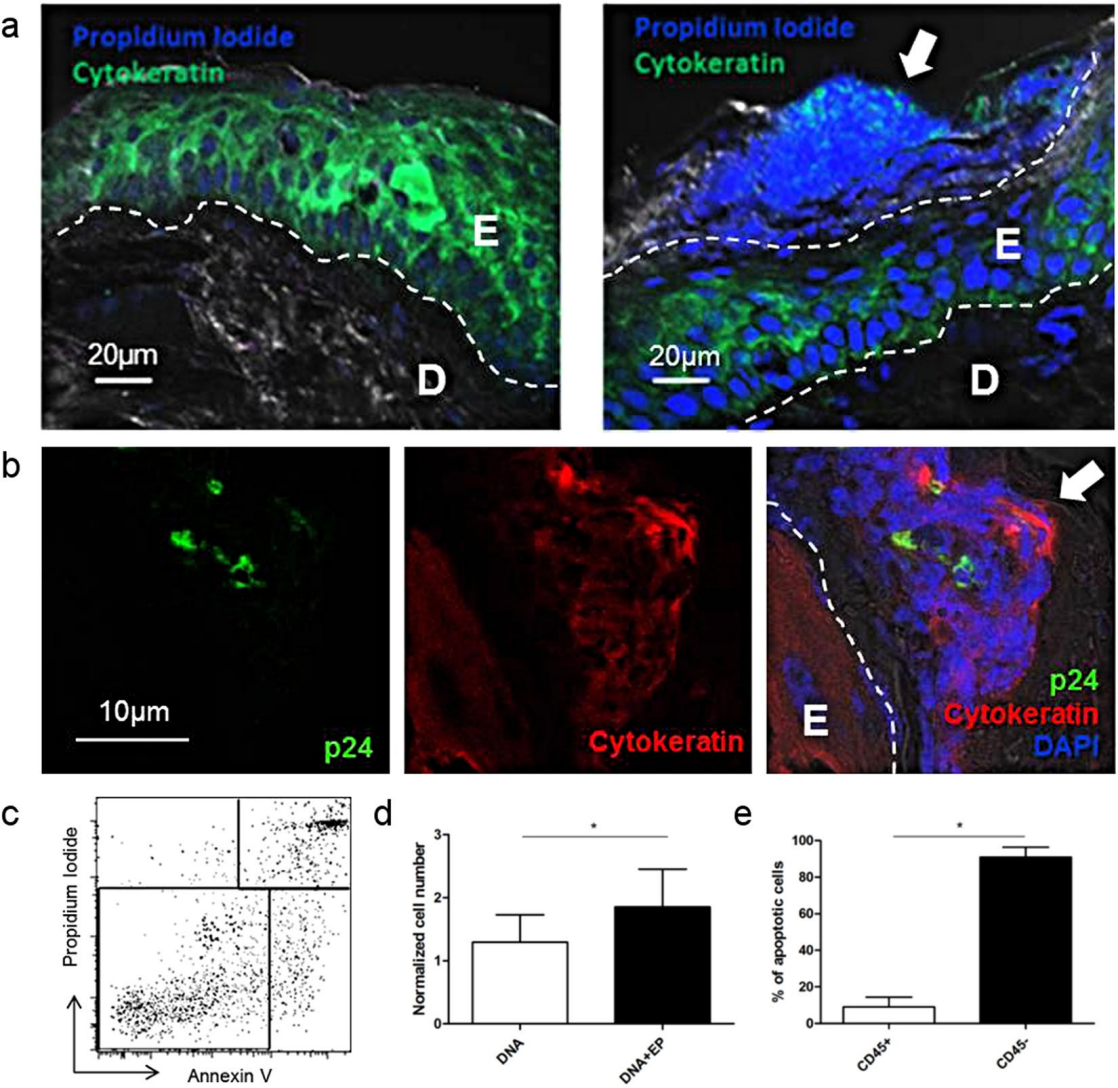
Vaccine antigen expression in damaged epidermal cells. (**a**) Representative images of frozen skin sections after PBS injection (left) or PBS with EP (right) (E: Epidermis; D: Dermis). (**b**) Skin section, 24 h after auxoGTU^®^-multiHIV vaccine injection with EP showing necrotic area in the stratum corneum and antigen expression (p24). (**c**) AnnexinV^+^Propidium Iodide^−^ apoptotic cell population gating in epidermal cells analyzed 24 h after DNA ± EP by flow cytometry. (**d**) The number of apoptotic cells 24 h after DNA ± EP normalized to the number of apoptotic cells quantified after PBS injection (n = 6) Wilcoxon test. (**e**) Proportion of CD45^+^/CD45^−^ apoptotic cells at 24 h after DNA ± EP (n = 4). Mann-Whitney test. Data are presented as mean ± SD; *p < 0.05, E: epidermis; D: dermis; EP: electroporation; arrows indicate damaged keratinocytes on the epidermal surface.

We observed damaged keratinocytes on the epidermal surface 24 h after PBS + EP (Fig. 5a). Interestingly, when EP was associated with DNA injection, vaccine antigen was detected in these dying keratinocytes (Fig. 5b). Furthermore, the number of apoptotic cells was significantly higher after vaccination with EP than after DNA alone (*p* = 0.0313) (Fig. 5c,d). These cells were mostly CD45^−^ (Fig. 5e) and may represent an additional stimulus for immune activation^12^.

### EP creates an early inflammatory environment

**Figure 6:**
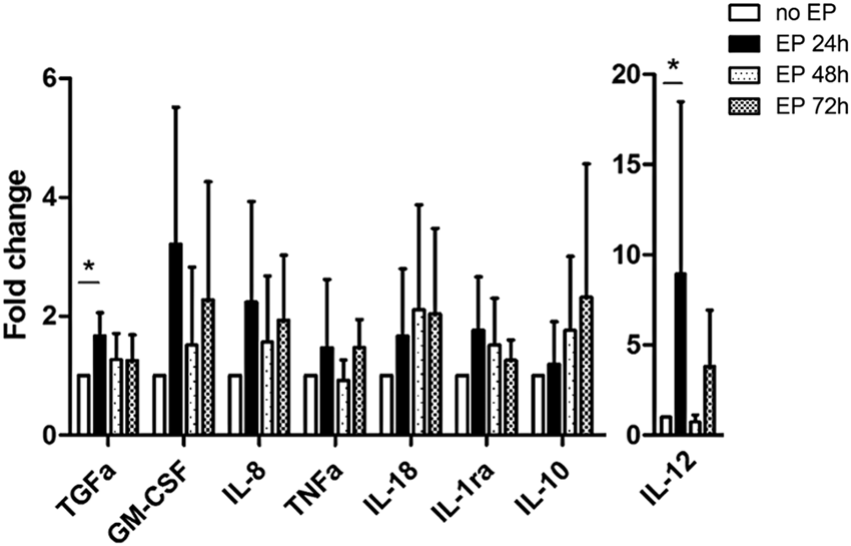
Cytokine production in the skin after EP. The cytokine concentrations were measured in the supernatants of skin biopsies after 6 h of incubation at 37 °C and 5% CO_2_. The values of the change fold were expressed as the ratio between the cytokine concentrations in the supernatant in each condition and the ones measured in the non-electroporated condition. Skin biopsies were performed 24, 48 and 72 hours after intradermal injection of PBS followed by EP. Kruskal-Wallis test (n = 5). Data are presented as mean ± SD; *p < 0.05, EP: electroporation.

Finally, we analyzed the cytokine microenvironment in supernatants of skin biopsies post-EP. The concentrations of TGF-α and IL-12 were significantly higher in skin 24 h after EP than in non-electroporated skin (*p* = 0.0274 and *p* = 0.0117) (Fig. 6). At this time point, the concentration of other pro-inflammatory (GM-CSF, IL-8, TNF-alpha, IL-18) and anti-inflammatory (IL-1ra, IL-10) cytokines, tended to be higher in electroporated skin than in non-electroporated skin.

## Discussion

Electroporation has long been used to facilitate the DNA access to the cell nucleus and its association with DNA vaccination clearly enhances the production of vaccine antigen in mice and large animal models^21–23^. However, it appears that high antigen expression is not the only factor that determines vaccine immunogenicity because electrical pulses applied prior to or simultaneously to DNA vaccination induce similar immune responses^24^. We used the auxo-GTU^®^-multiHIV plasmid to investigate the cellular effects of vaccination with EP. This plasmid is known for its ability to stimulate strong cellular responses which are significantly larger and more persistent when EP is used^19^. Consistent with other studies^17, 21, 23, 25, 26^ we confirmed that vaccine antigen was more strongly expressed when injection was combined with EP. Interestingly, EP seemed to affect the localization of transfected cells and they were predominantly present in the epidermis. High antigen availability in the epidermis may be beneficial due to the LC population localized in this skin layer. LCs are essential for T-cell responses, as shown by the induction of modified vaccinia virus Ankara (MVA)-specific CD8+ T cells^27^, and for antitumor cellular immunity^28^. *In vitro* experiments have revealed that LC are particularly effective at priming and cross-priming naïve CD8^+^ T cells^29^. However, there has been disagreement about LC functions *in vivo* and it is now accepted that their role depends on the quantity and the quality of immune signals^30^. EP *in vivo* promotes the migration of LCs out of murine skin explants^31^. In our experiments, DNA vaccination with EP caused morphological changes to LCs that have been previously associated with their migratory behavior^20^. In addition, the high motility of LCs, their interaction with DNA transfected cells, and their migration from the epidermis, shows that LCs are involved in the mechanism of vaccination with EP. Moreover, after EP, we found vaccine antigen in damaged keratinocytes on the skin surface. Thus, vaccination combined with EP provides an additional source of cell-associated antigen, which is easily accessible to surrounding cells for cross presentation^32^. Moreover, we showed that simple EP of macaque skin led to immune cell infiltration at the site of injection with a peak at 24 h (data not shown). These immune cell infiltrates have also been observed after intramuscular vaccination with EP in mice^33^. Recently, Markelc *et al*. demonstrated that electrical pulses increase blood vessel permeability, which is accompanied by leukocyte extravasation^34^. In our study, the overlap between the peak of antigen expression and APC recruitment may lead to robust antigen processing and presentation at the site of vaccination. Furthermore, the recruitment of lymphocytes to the skin (although non-specific to the antigen) may promote potent local responses after vaccine boost, as occurs during the recall of peripheral infection^35^.

We also identified several cytokines produced locally in response to electrical pulses without vaccination. IL-12 and GM-CSF have already been used as adjuvants to DNA vaccines^36, 37^. In particular, IL-12 promotes the accumulation of CD8^+^ T cells^38^ and the differentiation of naïve T cells to Th1^39^, whereas GM-CSF plays a central role in the development and maturation of dendritic cells and its overexpression in the epidermis induces both keratinocyte proliferation and apoptosis^40^. Interestingly, we found that the TGF-α concentrations were significantly higher in electroporated skin than in non-electroporated skin. This cytokine up-regulates TLR5 and TLR9 expression on keratinocytes and enhances the cell response to CpG DNA^41^ (CpG for cytosine–phosphate–guanine). Other pro-inflammatory cytokines induced by EP, participate in immune cell recruitment (IL-8)^42^ or in cell maturation and migration (TNFα, IL-18)^43, 44^. We also observed delayed production of anti-inflammatory cytokine IL-10 in electroporated skin, which may be important to limit inflammation caused by EP.

The analysis of subcutaneous tissues revealed that the effects of EP penetrate deep into the skin. We showed that when vaccination was performed with EP, the vector reached subcutaneous tissues, such as the subcutaneous muscle. In mouse skin, most transfected cells following EP are observed in the hypodermis and around the panniculus carnosus^16^. This muscle is involved in skin movements and is almost absent in humans, which may explain several interspecies differences regarding vaccine delivery through the skin, as occurs for some adeno-associated viral vectors which show skin muscle tropism after id injection^45^. Furthermore, recruited inflammatory cells in the panniculus carnosus may play an additional role in vaccine responses by creating a favorable environment for antigen processing and presentation.

Nevertheless, epidermal injury resulting from EP may induce the production of damage-associated molecular patterns and activate immune actors independent of vaccination. Indeed, uric acid and high mobility group protein B1 (HMGB1) have been detected in electroporated muscle with or without vaccination and have been associated with robust T cell responses to plasmid DNA antigens^46^. Tissue damage and inflammation depend on EP parameters such as the intensity, the duration of electrical pulses and the type of electrodes^47, 48^. The immune enhancer potency of cell death has been reported after vaccine delivery by micro-needles^49^ and this property is also being used to develop laser-based vaccine adjuvants^50^.

However, for clinical use, this immune stimulation due to electroporation should be counterbalanced by the discomfort which may occur with this procedure. In our study electroporation performed with needleless tweezers electrodes^51^, resulted in very slight and transitory superficial tissue injury. The same electroporation protocol has been previously tested on pig skin^19^ approved as biomedical model for human skin studies^52^.

Our findings demonstrate that EP is an effective vaccine delivery system and adjuvant. The application of EP to the skin broadens immune responses to vaccine in all skin layers and subcutaneous tissues by extending the delivery of antigen. Thus, vaccine protocols that promote local immune actors may abolish the need for chemical-based adjuvants.

## Materials and Methods

### Animals

Adult female cynomolgus macaques (*Macaca fascicularis*), imported from Mauritius and weighing 3–6 kg, were housed in CEA facilities (accreditation: C 92-032-02) and handled in accordance with European guidelines for nonhuman primates (NHP) care (EU Directive N 63/2010). This study was approved by the regional committee for animal care and use (Comité d’Ethique en Expérimentation Animale n° 44, AP N° 11_008). Animals were handled under sedation with an intramuscular injection of 10 mg/kg ketamine hydrochloride (Merial, France).

### Plasmids

The vaccine vector auxo-GTU^®^-multiHIV encodes a fusion protein composed of the full-length sequences of Rev, Nef, Tat, p17 and p24 proteins and a stretch of selected T cell epitopes from Pol and Env proteins^19^. For *in vivo* imaging studies, the expression cassette of HIV fusion protein was replaced with the DNA sequence encoding the luciferase protein and the enhanced green fluorescent protein (EGFP) protein.

### DNA vaccine injection

Skin was shaved at the site of injection. Each animal received id injections of 100 µl (1 mg/ml) of auxo-GTU^®^-multiHIV plasmid with or without EP. Phosphate-buffered saline (PBS) (100 µL) was injected as a control. EP was performed with a portable pulse generator (CUY21 EDIT; Nepa Gene, Ichikawa, Japan) and tweezer electrodes (6 pulses of 10 msec with output current 300–600 mA)^51^. For imaging studies of antigen expression, 100 µl (250 µg) of auxo-GTU^®^-Luc-EGFP plasmid was injected.

### Bioluminescence imaging

Antigen expression was measured between 1 h and 24 days after the injection of auxo-GTU^®^-Luc-EGFP plasmid with or without electroporation. Before imaging, macaques were injected intraperitoneally with 42 mg/kg of luciferin solution (Promega Corporation, Madison, Wisconsin). The PhotonImager^TM^ system (Biospace Lab, France) was used to acquire photonic emissions (photons/s/cm^2^) 30 min after the injection of luciferin. Luciferase expression was quantified in regions of interest manually drawn around the site of injection and was expressed as the area under the curve (AUC) after subtraction of the background signal measured on untreated skin.

### *In vivo* fibered confocal fluorescence microscopy

Antigen (EGFP) expressing skin cells and epidermal APC were monitored after vaccination by *in vivo* fibered confocal microscopy at 488 nm (Cellvizio^®^, Mauna Kea Technologies, France). For epidermal APC imaging, the monoclonal antibody (Ab) anti-HLA-DR (clone L243, Ozyme, France) was amine-labeled with the Fluoprobe 490 kit (Interchim, France). Five µg of fluorescent Ab was injected id into the middle of each vaccination site, 5 min and then 49 h after vaccination. Epidermis imaging was performed with the probe S-1500, which covers a 300 µm circular diameter in a focal plane and provides images from the skin surface, with a slice thickness of 15 µm and a lateral resolution of 3.3 µm. The Z-1800 probe was used for dermal imaging. This probe is 1.8 mm in diameter and acquires fluorescent signals emitted from 100 µm in depth, with a lateral resolution of 3.5 µm. Images were collected from at least 10 different areas per injection site from three independent experiments (n = 30). Images were analyzed with ImageJ 1.6 software (National Institute of Mental Health, Bethesda, USA).

### *Ex vivo* imaging on skin explants

Microscopic studies of tissue explants were performed as described previously^53^. Briefly, skin biopsies were performed 24 hours after the injection auxo-GTU^®^-Luc-EGFP plasmid and 2 hours after the *in vivo* id injection of anti-HLA-DR-AF700 Ab (Ozyme, France). Fat tissue was removed and each biopsy was cut into two equal parts and placed in a 6-well plate (MatTek Corporation, Ashland, USA) in contact with complete culture medium to visualize dermal and epidermal skin layers. Images were acquired with a Plan Fluor 20x DIC objective (NA: 0.45) on a Nikon A1R confocal fast laser scanning system (Nikon Corporation, Japan) equipped with a thermostatic chamber (37 °C; 5% CO_2_). Images were recorded with a high-speed resonant scanner every 10 minutes for 22 hours. Volocity software (Perkin Elmer, Waltham, USA) was used to reconstruct three-dimensional images and to assess cell motility (velocity, displacement and confinement ratio).

### Immunohistofluorescence of skin sections

Skin biopsies were performed 24 hours post-injection and embedded in optimal cutting temperature compound (OCT) and frozen in liquid nitrogen. Ten micrometer sections were fixed in 4% of Paraformaldehyde (PFA) for 15 min at room temperature. Tissue was permeabilized with Triton X-100 (0.3%), Bovine serum albumin (BSA) (1%) and goat serum (1%). Skin sections were incubated overnight at 4 °C with 10 µg/ml of anti-GFP-AF488 Ab (Invitrogen, France) or anti-p24 Ab (kind gift from B. Verrier, UMR 5086 CNRS/UCBL, Lyon, France) to detect the expression of vaccine antigens in animals injected with the auxo-GTU^®^-Luc-EGFP or the auxo-GTU^®^-multiHIV plasmid, respectively. For keratinocyte staining, an additional incubation for 2 hours was performed with an anti-cytokeratin Ab (clone AE1/AE3, Dako, France). Purified antibodies were labeled with the Zenon^®^ labeling kit (Molecular Probes, Invitrogen). The cell nucleus was stained with 4′,6-diamidino-2-phenylindole (DAPI) or Propidium Iodide (Invitrogen, France).

### Flow cytometry

To characterize skin immune cells, 8 mm-biopsies were taken from the site of vaccination. The skin (dermis and epidermis) was separated from the cutaneous muscle (panniculus carnosus) and hypodermis fat tissues, and each tissue was mechanically dissociated (GentleMACS^TM^ dissociator, Miltenyi, France). Cells were then filtered through 70 µm nylon mesh after enzymatic treatment with 2 mg/ml of Collagenase D (Roche Diagnostics, France) for 30 minutes at 37 °C. The LIVE/DEAD Fixable Blue Dead Cell Stain Kit (Invitrogen, France) was used to label dead cells. Cell surface staining was performed with the following antibodies: anti-CD45 (clone DO58-1283, BD, France), HLA-DR (clone G46-6, BD, France), anti-CD66abcd (clone TET2, Miltenyi Biotec, France), anti-CD3 (clone SP34-2, BD, France), anti-CD163 (clone GHI/61, Ozyme, France), anti-CD14 (M5E2, BD, France). Apoptotic cells were stained with AnnexinV and Propidium Iodide (Invitrogen, France) according to the manufacturer’s instructions. Data were acquired on LSR Fortessa (BD Biosciences, Le Pont de Claix, France) and analyzed with FlowJo software 9.4.11 (Tree Star, Ashland, OR).

### Multiplex assay

Supernatants were collected after an 18 h incubation of skin biopsies in complete media. High-sensitivity cytokine detection was performed by Milliplex^®^ assay (Merck Millipore, Billerica, Massachusetts) to analyze the production of Granulocyte-Macrophage Colony Stimulating Factor (GM-CSF), interferon (IFN)-γ, interleukine (IL)-1β, IL-1ra, IL-2, IL-6, IL-8, IL-10, IL-12/23(p40), IL-17, Monocyte Chemoattractant Protein (MCP)-1, macrophage inflammatory protein (MIP)-1α, MIP-1β, Transforming Growth Factor (TGF)-α, tumor necrosis factor (TNF)-α, IL-18.

### Statistical analysis

Data are reported as means ± standard deviation (SD) and analyzed with GraphPad Prism version 5.0 (Graph-Pad Software Inc, La Jolla, CA) with the appropriate non-parametric ANOVA (Friedman or Kruskal-Wallis) or t-tests (Wilcoxon or Mann-Whitney).

## References

[1] Kutzler MA, Weiner DB (2008). DNA vaccines: ready for prime time?. Nat Rev Genet.

[2] Lu S, Wang S, Grimes-Serrano JM (2008). Current progress of DNA vaccine studies in humans. Expert Rev Vaccines..

[3] Laddy DJ (2008). Heterosubtypic protection against pathogenic human and avian influenza viruses via *in vivo* electroporation of synthetic consensus DNA antigens. PLoS One..

[4] Blazevic V (2006). Induction of human immunodeficiency virus type-1-specific immunity with a novel gene transport unit (GTU)-MultiHIV DNA vaccine. AIDS Res Hum Retroviruses.

[5] Zhou W (2002). Multiple RNA splicing and the presence of cryptic RNA splice donor and acceptor sites may contribute to low expression levels and poor immunogenicity of potential DNA vaccines containing the env gene of equine infectious anemia virus (EIAV). Vet Microbiol..

[6] Sajadian A (2014). Comparing the effect of Toll-like receptor agonist adjuvants on the efficiency of a DNA vaccine. Arch Virol..

[7] Barouch DH (2002). Potent CD4+ T cell responses elicited by a bicistronic HIV-1 DNA vaccine expressing gp120 and GM-CSF. J Immunol..

[8] Okada E (1997). Intranasal immunization of a DNA vaccine with IL-12- and granulocyte-macrophage colony-stimulating factor (GM-CSF)-expressing plasmids in liposomes induces strong mucosal and cell-mediated immune responses against HIV-1 antigens. J Immunol..

[9] Weiss WR (1998). A plasmid encoding murine granulocyte-macrophage colony-stimulating factor increases protection conferred by a malaria DNA vaccine. J Immunol..

[10] Parsania M (2010). Evaluation of apoptotic and anti-apoptotic genes on efficacy of DNA vaccine encoding glycoprotein B of Herpes Simplex Virus type 1. Immunol Lett..

[11] Bergmann-Leitner ES, Leitner WW, Duncan EH, Savranskaya T, Angov E (2009). Molecular adjuvants for malaria DNA vaccines based on the modulation of host-cell apoptosis. Vaccine.

[12] Brave A (2010). Induction of HIV-1-specific cellular and humoral immune responses following immunization with HIV-DNA adjuvanted with activated apoptotic lymphocytes. Vaccine.

[13] Sardesai NY, Weiner DB (2011). Electroporation delivery of DNA vaccines: prospects for success. Curr Opin Immunol.

[14] Song JM (2012). DNA vaccination in the skin using microneedles improves protection against influenza. Mol Ther..

[15] Heller R, Cruz Y, Heller LC, Gilbert RA, Jaroszeski MJ (2010). Electrically mediated delivery of plasmid DNA to the skin, using a multielectrode array. Hum Gene Ther..

[16] Roos AK (2009). Skin electroporation: effects on transgene expression, DNA persistence and local tissue environment. PLoS One..

[17] Drabick JJ, Glasspool-Malone J, King A, Malone RW (2001). Cutaneous transfection and immune responses to intradermal nucleic acid vaccination are significantly enhanced by *in vivo* electropermeabilization. Mol Ther..

[18] Bellard E (2012). Intravital microscopy at the single vessel level brings new insights of vascular modification mechanisms induced by electropermeabilization. J Control Release..

[19] Martinon F (2009). Persistent immune responses induced by a human immunodeficiency virus DNA vaccine delivered in association with electroporation in the skin of nonhuman primates. Hum Gene Ther.

[20] Pearton M (2010). Changes in human Langerhans cells following intradermal injection of influenza virus-like particle vaccines. PLoS One..

[21] Babiuk S (2003). Needle-free topical electroporation improves gene expression from plasmids administered in porcine skin. Mol Ther..

[22] Hirao LA (2008). Intradermal/subcutaneous immunization by electroporation improves plasmid vaccine delivery and potency in pigs and rhesus macaques. Vaccine.

[23] Simon AJ (2008). Enhanced *in vivo* transgene expression and immunogenicity from plasmid vectors following electrostimulation in rodents and primates. Vaccine..

[24] Peng B, Zhao Y, Xu L, Xu Y (2007). Electric pulses applied prior to intramuscular DNA vaccination greatly improve the vaccine immunogenicity. Vaccine.

[25] Guo S (2011). Electro-gene transfer to skin using a noninvasive multielectrode array. J Control Release.

[26] Guo S, Israel AL, Basu G, Donate A, Heller R (2013). Topical gene electrotransfer to the epidermis of hairless guinea pig by non-invasive multielectrode array. PLoS One..

[27] Liard C (2012). Intradermal immunization triggers epidermal Langerhans cell mobilization required for CD8 T-cell immune responses. J Invest Dermatol.

[28] Stoitzner P (2008). Tumor immunotherapy by epicutaneous immunization requires langerhans cells. J Immunol..

[29] Klechevsky E (2008). Functional specializations of human epidermal Langerhans cells and CD14+ dermal dendritic cells. Immunity..

[30] Romani N, Brunner PM, Stingl G (2012). Changing views of the role of Langerhans cells. J Invest Dermatol.

[31] Zhao YL (2006). Induction of cytotoxic T-lymphocytes by electroporation-enhanced needle-free skin immunization. Vaccine..

[32] Fonteneau JF (2003). Characterization of the MHC class I cross-presentation pathway for cell-associated antigens by human dendritic cells. Blood..

[33] Liu J, Kjeken R, Mathiesen I, Barouch DH (2008). Recruitment of antigen-presenting cells to the site of inoculation and augmentation of human immunodeficiency virus type 1 DNA vaccine immunogenicity by *in vivo* electroporation. J Virol..

[34] Markelc B (2012). *In vivo* molecular imaging and histological analysis of changes induced by electric pulses used for plasmid DNA electrotransfer to the skin: a study in a dorsal window chamber in mice. J Membr Biol.

[35] Wakim LM, Gebhardt T, Heath WR, Carbone FR (2008). Cutting edge: local recall responses by memory T cells newly recruited to peripheral nonlymphoid tissues. J Immunol..

[36] Hirao LA (2008). Combined effects of IL-12 and electroporation enhances the potency of DNA vaccination in macaques. Vaccine..

[37] Rodriguez AM (2012). IL-12 and GM-CSF in DNA/MVA immunizations against HIV-1 CRF12_BF Nef induced T-cell responses with an enhanced magnitude, breadth and quality. PLoS One..

[38] Kim MT, Harty JT (2014). Impact of Inflammatory Cytokines on Effector and Memory CD8+ T Cells. Front Immunol..

[39] Hsieh CS (1993). Development of TH1 CD4+ T cells through IL-12 produced by Listeria-induced macrophages. Science..

[40] Breuhahn K (2000). Epidermal overexpression of granulocyte-macrophage colony-stimulating factor induces both keratinocyte proliferation and apoptosis. Cell Growth Differ.

[41] Miller LS (2005). TGF-alpha regulates TLR expression and function on epidermal keratinocytes. J Immunol..

[42] Baggiolini M, Clark-Lewis I (1992). Interleukin-8, a chemotactic and inflammatory cytokine. FEBS Lett.

[43] Cumberbatch M, Dearman RJ, Antonopoulos C, Groves RW, Kimber I (2001). Interleukin (IL)-18 induces Langerhans cell migration by a tumour necrosis factor-alpha- and IL-1beta-dependent mechanism. Immunology..

[44] Lebre MC (2003). Double-stranded RNA-exposed human keratinocytes promote Th1 responses by inducing a Type-1 polarized phenotype in dendritic cells: role of keratinocyte-derived tumor necrosis factor alpha, type I interferons, and interleukin-18. J Invest Dermatol.

[45] Galeano M (2003). Adeno-associated viral vector-mediated human vascular endothelial growth factor gene transfer stimulates angiogenesis and wound healing in the genetically diabetic mouse. Diabetologia.

[46] Barbon CM (2010). *In vivo* electroporation enhances the potency of poly-lactide co-glycolide (PLG) plasmid DNA immunization. Vaccine..

[47] Lin F (2012). Optimization of electroporation-enhanced intradermal delivery of DNA vaccine using a minimally invasive surface device. Hum Gene Ther Methods.

[48] Breton M, Mir LM (2012). Microsecond and nanosecond electric pulses in cancer treatments. Bioelectromagnetics..

[49] Depelsenaire AC (2014). Colocalization of cell death with antigen deposition in skin enhances vaccine immunogenicity. J Invest Dermatol.

[50] Chen X, Wang J, Shah D, Wu MX (2013). An update on the use of laser technology in skin vaccination. Expert Rev Vaccines.

[51] Adam L, Le Grand R, Martinon F (2014). Electroporation-mediated intradermal delivery of DNA vaccines in nonhuman primates. Methods Mol Biol.

[52] Hengge UR, Walker PS, Vogel JC (1996). Expression of naked DNA in human, pig, and mouse skin. J Clin Invest.

[53] Salabert N (2016). Intradermal injection of an anti-Langerin-HIVGag fusion vaccine targets epidermal Langerhans cells in nonhuman primates and can be tracked *in vivo*. Eur J Immunol.

